# 

**Published:** 1900-09-01

**Authors:** 


### NOTICE TO CORRESPONDENTS.

**All MS., Letters, Books for Review, and other matters intended for the Editor should be addressed The Editor, "The Hospital," The Hospital Building, 28 & 29, Southampton Street, Strand, W.C. The Editor cannot undertake to return rejected MS., even when accompanied by stamped directed envelope.**

**Notice to Advertisers and Subscribers.**

**All Advertisements, Orders for Copies of The Hospital, and Business Communications should be addressed to THE MANAGER (Not to the Editor), The Hospital, The Hospital Building, 28 & 29, Southampton Street, Strand, London, W.C.**

**The Cost of Subscription, post free, is as follows:—Per week, 2½d.; per quarter, 2s. 8d.; per half-year, 5s. 3d.; per annum, 10s. 6d. Foreign:—Per annum, 15s. 2d., payable, in all cases, in advance.**

**NOTICE.—Vol. XXVII. of "The Hospital" (October, 1899, to April, 1900), in handsome cloth binding, is now on sale at the Office of this Journal, price 7s. 6d. Cases for binding the Numbers contained in this Volume 1s. 6d. Index to Vol. XXVII., 2½d., post free.**

**The Monthly Part for September is now ready. Price 6d., by post 8d.**

### BOOKS RECEIVED.

Jarrold and Sons.

"Bunce, the Bobby, and the Broads: A Holiday Yarn." By Fritz Zorn.

Milner and Longbottom, Bradford.

"Urine Testing for Nurses." By J. Beard, M.R.C.S., L.R.C.P.

J. and A. Churchill.

"A Short Practice of Gynæcology." By Henry Jellett, B.A., M.D.

Baillière, Tindall, and Cox.

"Electricity in Gynæcology." By Richard Cowen, L.R.C.S.I., L.R.C.P.I.

Henry Kimpton.

"The Health Resorts of Europe." By Thomas Linn, M.D. Eighth year.

G. W. Bacon and Co.

"China and the Present Crisis." By R. L. Jeafferson, F.R.C.S. With map and glossary.

### Scraps and Gleanings.

The sad accident which occurred at Charing Cross Hospital last week, in which a girl lost her life through the upsetting of some beeswax and turpentine melting on a stove, should serve to warn housekeepers that there are many excellent polishing preparations which are far safer and more economical.

At the Paris Exhibition two Grands Prix have been awarded to the Pasteur filter, one to Monsieur Chamberland in the class of General Hygiene, and one to the manufacturers in the class of Military Hygiene. Messrs. Defries and Sons inform us that no Grand Prix has been awarded to any other filter.

A garden fête and sale of work was recently held at Mr. Holbrook Gaskell's estate at Woolton Wood in aid of the Liverpool Country Hospital for Chronic Diseases of Children. This institution commenced work last November with 20 beds, but this number has already been found inadequate to meet the demands for admission; and it was for the purpose of raising sufficient funds to purchase a site for a hospital to contain a larger number of beds that this fête was organised. The sale was opened by Mr. Gaskell, who promised a contribution of £1,000, and another donation of £500 by Mr. Andrew Gibson was announced. An interesting collection of Japanese pictures was lent for the occasion by Colonel Montgomery.

### The Student of Medicine.

### APPOINTMENTS.

A. Bisset, M.B., Ch.B., appointed Resident Physician and Surgeon to the Aberdeen Royal Infirmary. F. W. E. Courtenay, L.R.C.P., L.R.C.S.Edin., L.F.P.S.Glasg., appointed Surgeon on board the Allan Mail steamship "Numidian," for Canada. C. E. P. Forsyth, M.B., Ch.B., appointed Assistant Medical Officer, Metropolitan Asylums Board, to the Eastern Hospital, Homerton, N.E. T. E. Frazer-Toovey, L.R.C.P., L.R.C.S.Edin., L.F.P.S.Glasg., L.M.Rotunda, appointed House Surgeon to the Teignmouth Hospital, S. Devon. A. M. Rattray, M.B., C.M. Edin., appointed Senior Assistant Medical Officer to the Newcastle City Asylum, Gosforth. G. H. Sutherland, M.A., M.D., M.R.C.P., appointed Honorary Physician to the City Orthopædic Hospital. W. Sykes, M.D., appointed Honorary Medical Officer to the Woking Victoria Cottage Hospital.

**THE MENOPAUSE AND ITS DISORDERS.** With Chapters on Menstruation. By A. D. LEITH NAPIER, M.D., M.R.C.P., late Editor of the *British Gynæcological Journal*. Royal 8vo., cloth gilt, illustrated by a series of Original Photo-micrographs and many Illustrations in the Text, 7s. 6d. net.

London: The Scientific Press, Ltd., 28 & 29, Southampton Street, Strand, W.C.

### THE NURSE'S REPORT BOOK.

Foolscap 4to., Black cover, **6d.** net, post free; or 12 for **4s. 6d.**, post free.
**Compiled by K. H. (Cert.)**

H. J. GLAISHER, 57, Wigmore Street, Cavendish Square, W.

